# Management of Hepatic Rupture Diagnosed after an Emergency Cesarean Section

**DOI:** 10.1155/2014/312938

**Published:** 2014-08-31

**Authors:** Gianluca Raffaello Damiani, Viviana Merola, Mario Barnaba, Stefano Landi, Gennaro Cormio, Antonio Pellegrino

**Affiliations:** ^1^Department of Obstetrics and Gynecology, Alessandro Manzoni Hospital, Dell'Eremo Street 11, 23900 Lecco, Italy; ^2^Department of Obstetrics and Gynecology, University of Bari, 70124 Bari, Italy; ^3^Department of Obstetrics and Gynecology, Hospital of Sondrio, 23100 Sondrio, Italy

## Abstract

A careful management of hepatic capsular rupture, with massive hemoperitoneum which occurred 14 hours after an emergency cesarean section at 36 weeks of gestation, is meticulously reported. The grade of hepatic involvement varies from minor capsular laceration to extensive parenchymal rupture. Our management involved a combination of surgical interventions and aggressive supportive care. The patient was discharged after 53 days and 4 laparotomies and an unsuccessful attempt of superselective artery embolization. Ultrasound after 40 days from the last surgery showed uniform hepatic parenchyma free of focal lesions. Due to the rarity and the unpredictability nature of this devastating event we believe necessary to report our experience, reinforcing the importance of the postsurgery management.

## 1. Introduction

Spontaneous hepatic rupture (HR) is an infrequent and life-threating condition of pregnancy that is virtually exclusively associated with severe preeclampsia (PE) or HELLP syndrome (HS) with an incidence of 1 per 2000 patients [1]. The pathophysiology of HR includes the sequence of hypertension, vasospasm, hemolysis, fibrin deposition and platelets aggregation, sinusoidal obstruction, vascular congestion, and hepatic ischemia/infarction. In the present case intraparenchymal and subcapsular hemorrhage developed, resulting in HR.

Fatality rates are high despite the successes in hepatic surgery and critical care unit assistance. The crucial pivotal points are the atypical clinical presentation of PE or HS, taking into account that HR can occur without clinical signs or warning.

Prompt radiologic and laboratory evaluations are essential and may reduce the associated morbidity and mortality. Accumulation of these rare reports is important for current obstetrics.

## 2. Case

A 32-year-old nulliparous woman, after in vitro fertilization (IVF), was referred to our department at 35^+5^ weeks' gestation presenting elevated blood pressure (BP) without proteinuria. The patient's BP was occasionally borderline since the 27th week of gestation. On admission BP was 170/90 mmhg, proteinuria was 0.59/24 g/h, ast was 16 and alt was 14 u/L, uric acid was 6.5 mg/dL, platelets were 260 10^9^/L, and d-dimero was 400 mg/L. Uterine and middle cerebral Doppler were within normal ranges. The patient was without any complaints. She denied any occurrence of headache, change in vision, or epigastric pain. Her personal history was unremarkable. After initial response to Nifedipina, Labetalol was administered (15 mL/h). Subsequent BP values were within normal limits. Betamethasone was administered for prevention of RDS. Four days after admittance, the patient complained of mild epigastric pain, accompanied by vomiting. Labor was induced using Foley transcervical catheter. Labetalol was increased to 25 mL/h.

After 12 h from labor induction, lab tests presented a slight increase in liver enzymes, while platelets were stable (Table 1). Proteinuria was 3.55 g/24 h. Eight h later, there was a significant increase of aspartate-alanine aminotransferase (ast 204 u/L, alt 165 u/L) and lactic dehydrogenase (ldh 500 u/L). BP was 160/110, the patient complained of headache and moderate epigastric pain. Cesarean section (CS) was performed owing to worsening clinical and laboratory parameters and a female infant was born with Apgar scores of 9 and 10 after 1 and 5 min, respectively. The CS was uncomplicated and there were no findings of hemoperitoneum.

Four h later, the uterus was contracted and the onset of diuresis was optimal (200 mL/h). Proteinuria was 7.78 g/24 h. 18 h after CS, the patient complained of general malaise and developed marked abdominal distension. Ultrasound showed inferior vena cava diameter of 1.3 mm in M-mode and free fluid in abdomen suggestive of hemoperitoneum. Blood sample revealed a severe anemic situation (hb 6.6 mg/dL, ht 20.6%, and diuresis 15 mL/h).

**Figure 1:**
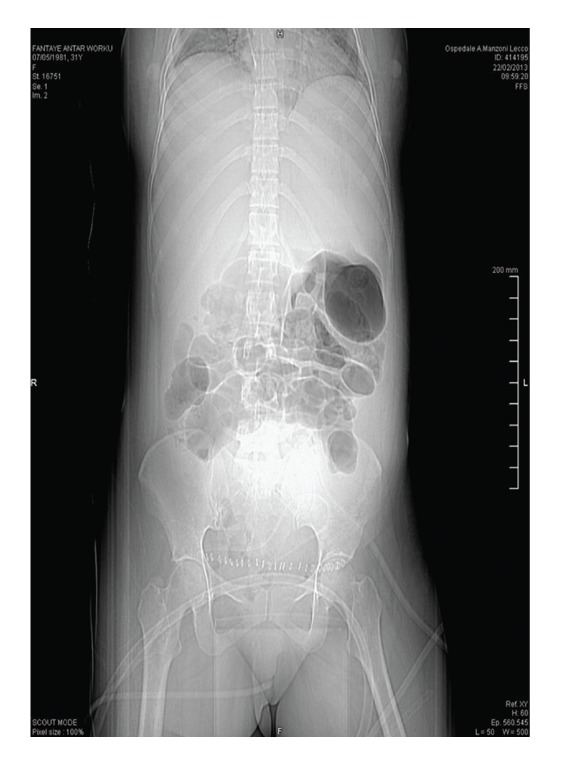
*CT scan:* intraparenchymal hepatic hematoma with active bleeding from the right and left ramus of hepatic artery. A concomitant subcapsular hematoma with an extra capsular caudal extension in the paracolic gutter.

The patient was taken to the operating room for an explorative laparotomy. Intraoperative findings included massive hemoperitoneum, about 800 mL of fresh blood was aspirated, and uterine incision was repaired, and resulted hemostatic. Superior abdomen was explored and no active bleeding was evidenced. Four units of fresh blood (1000 mL), 6 units of fresh frozen plasma (3600 mL), and 1 unit of platelets were transfused. Magnesium sulphate and labetalol were infused at 25 mL/h (1 g/h). 14 h later, abdominal ultrasound revealed a voluminous hematoma which interested the upper quadrant, right hypochondrium. A CT scan revealed a 14 cm intraparenchymal hepatic hematoma with active bleeding from the right and left ramus of hepatic artery. Another subcapsular hematoma of 11 cm which was extended into the paracolic gutter was evidenced (Figure 1). There was a wide area of moire in hepatic parenchyma due to an ipoperfusion caused by extrinsic compression. Free fluid was present in the Douglas space.

**Figure 2:**
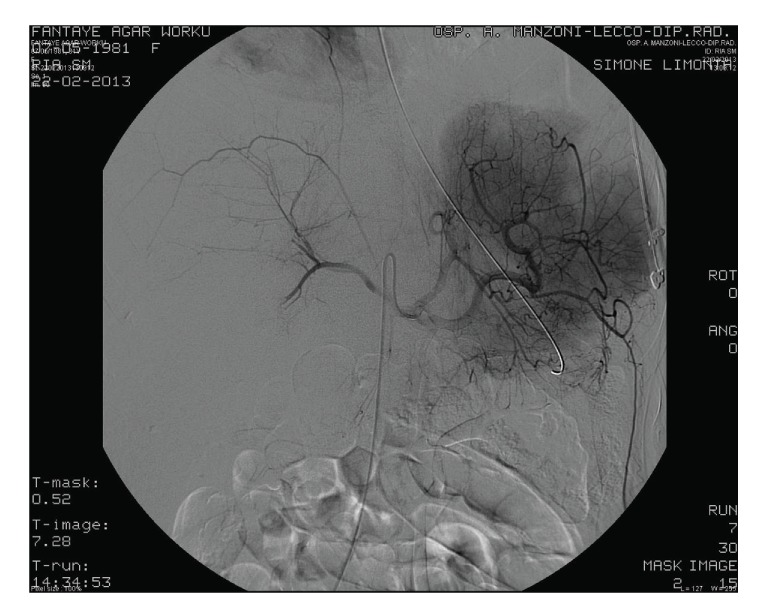
Superselective embolization of hepatic right artery.

Superselective embolization of hepatic right artery was performed, according to Seldinger technique (5 Fr Simmons 2 catheter), with microcatheter Progreat (Terumo-0.035) and introduction of spongostan; also left artery was treated (Figure 2).

CT scan showed outbreaks of bleeding in both of the hepatic lobes. Due to the hepatic ischemic insufficiency after embolization, an explorative laparotomy with xifopubic incision was performed in order to decompress the portal system: a voluminous hematoma on the dome of the liver was confirmed. The falciform and round ligaments were sectioned. At the manual examination a wide laceration of Glisson's capsule was noticed. Direct hemostasis was not achievable, because the liver was edematous. Evacuation of the hematoma compressing the left hepatic lobe was performed. Hematoma on the right lobe was not removed, where the original lesion was located, in order to avoid severe hemorrhage. Portal venous flussimetry resulted was normal, and a perihepatic packing was applied (14 gauzes). A Bogota bag was necessary in effort to reduce the intra-abdominal pressure to restore system perfusion. In postsurgery course there was an improvement of the conditions of the patient, with a decrease of transaminases. The surgical revaluation was programmed the following day. Remotion of further clots was performed and no active bleeding was evidenced.

A new packing (8 gauzes) and a steridrape in contact with hepatic surface was applied; 4 drainages were fixed in subdiaphragmatic region. Three days later (8 days after CS) laparotomy was performed to remove further clotting and gauzes. For the increased intra-abdominal pressure, it was necessary to insert a dual mesh and perform a partial closure of the abdominal fascia. Pleural effusion resulted in the chest X-ray with parenchymal dysventilation. Over the course of the next 6 days hyperpyrexia was present and a CT was programmed: the parenchimografia was improved and the hematoma was organized under the rectum muscles (18 × 6 × 25 cm). Bilateral pleural effusion was revealed, conditioning the atelectasis of the right inferior lobe. Thoracentesis was performed with evacuation of serous amber liquid (500 cc). Cultural exam was negative.

On the 14th day after CS, a percutaneous drainage was inserted in the right hypochondrium towards the accumulation of blood. 1400 cc of serous hematic liquid was aspirated. Ultrasound showed a decrease of hematoma with thickness of 3 cm. Bilateral pleural effusion was in resolution. Hepatic drainage was removed and a retraction of 5 cm of pleural drainage was made and removed 48 h later. On the 29th day after CS abdomen, ultrasound showed 2 residual areas of hematoma (thickness of 10 mm and 26 mm) in subdiaphragmatic region. Due to the presence of hyperpyrexia, teicoplanin and imipenem were administered for 10 days with discharge of the patient. An ultrasound performed 15 days later (45 days from the last surgery) shows uniform hepatic parenchyma free of focal lesions.

## 3. Discussion

Spontaneous liver hemorrhage with formation of subcapsular hematomas and rupture of Glisson's capsule is a rare but often lethal complication of pregnancy. The risk of HR is not reduced after labor, but it may be present 24–48 h after labor and the time between PE and HR may be hours, days, or weeks [2].

In the current case, HR, that occurred 15 h after CS, was so severe that four laparotomies were needed. In total 14 units of fresh blood (3500 mL), 9 units of fresh frozen plasma (5400 mL), and 2 units of platelets were transfused to the patient. Patients developing PE and especially HS require close monitoring owing to an insidious course that can lead to a misdiagnosis or confusion with other gastroenteric diseases. The affected patients may present with a sudden onset of abdominal pain, especially the right upper quadrant pain that radiates to right shoulder, nausea, vomiting, abdominal distension, and hypertension or hypovolemic shock. The rupture most frequently involves the right hepatic lobe, which represents 77% of the cases [3]. When the subcapsular liver hematoma is limited, conservative mamagement is possible, with an appriopate support [4]. The postoperative development was, in the main, not complication free. It is difficult to manage the postsurgery course due to slow hepatic healing, bilateral pleural effusion, and hyperpyrexia, due to residual clots. Our management has included decompressive laparotomies, hepatic packing, and partial abdominal closure technique to postpone definite closure until predisposing factors causing elevation of intra-abdominal pressure.

The optimal therapy can be debated, including hepatic artery ligation, hepatorraphy, collagen sponges, fibrin glue, aron laser, and recombinant factor VIIa [5]. According to our knowledge no correlation was between liver tests and HR, in order to prevent hepatic lesions. Changes of liver tests became increasingly abnormal, only when the hepatic rupture had already occurred. Values of ldh, ast, and alt levels were, respectively, 1,960 U/L, 1,745 U/L, and 1,196 U/L near the time of HR. In our opinion to diagnose HR and plan an adequate clinic management, patients with PE and HS with associations of symptoms as epigastric and right upper quadrant pain should undergo ultrasound or CT scans especially in the first 24 hours of the puerperium and when liver test function >800 U/L (ldh, ast, alt). In our department we prefer the surgical approach of liver packing and hepatic embolization due to the lowest mortality rate.

We have used embolization as the first line treatment, once the patient was hemodynamically stable. In some cases, HR could develop without classic signs of PE, but when a low platelet count and elevated ast/alt levels are present, it is difficult to differentiate whether the abnormal laboratory findings are the results of atypical PE or HS or are the result of liver hematoma [6].

The best approach is still not established and a unique strategy cannot be defined.

In summary, a diagnosis of HR should be considered when there is a sudden onset of hypotension and acute anemia in a patient with pregnancy-induced hypertension. This type of evaluation should lead to prompt diagnostic tests for HR, followed with adequate hemodynamic support and regular imaging.

## Figures and Tables

**Table 1:** Lab findings from the first day to the initial decrease of liver test 27 hours later after 2° laparotomy.

	1°	2°	3°	4°	12 h later	8 h later (1 h before CS)	4 h after CS	10 h later	5 h later	3 h later	3 h after 1°lp	3 h later	5 h later	3 h after 2°lp	12 h later	12 h later
Hb g/dL	11.6	10.5	11	11.9	11.9	12.5	12.9	11.9	9.1	6.6	9.2	8.1	8.6	9.9	9.3	12
Ht%	34.6	31.1	33.4	35.8	35.9	37.6	37.7	35	28.4	20.2						
Plt 10^9^/L	261	212	253	282	245	190	139	96	83	104	68	70	78	38	60	40
Ldh U/L	231	229	221	242	279	500	559	686	549	668	1888	1960	2003	3741	4978	4835
Total bilirubin Mg/dl	0.14	0.15	0.15	0.16	0.20	0.38	0.48	0.64	0.79	0.98	1.56 0.50d 1.06i		1.97 o.98 direct bilirubin 0.99i		3.23	
Ast U/L	21	20	20	43	70	204	248	261	219	342	1341	1745	1894 indirect	4123	6160	4500
Alt U/L	19	17	16	49	67	165	189	210	191	294	997	1196	1232	2237	2734	1400
Pro g/dL	6.6	6.6	6.2	6.3	6.6	6.6	6.3	6.1	4.9							
Azo Mg/dL	19	19	20	30	29	29	28			33						
Inr	0.94	0.92	0.92	0.88	0.88	0.89	0.91	0.97	1.02							
Ptt (s)	30 s/1.07	29/1.01	29/1.02	27/ 0.94	27/0.91	28/0.88	31/0.92	39/1.38								
Fibrinogen Mg/dL	378	365	370	286	279	311	407	424	303							
